# First Report of a Peroxiredoxin Homologue in Jellyfish: Molecular Cloning, Expression and Functional Characterization of CcPrx4 from *Cyanea capillata*

**DOI:** 10.3390/md12010214

**Published:** 2014-01-09

**Authors:** Zengliang Ruan, Guoyan Liu, Beilei Wang, Yonghong Zhou, Jia Lu, Qianqian Wang, Jie Zhao, Liming Zhang

**Affiliations:** 1 Department of Marine Biotechnology, Faculty of Naval Medicine, Second Military Medical University, Shanghai 200433, China; E-Mails: ruanzengliang@qq.com (Z.R.); lgy_laurie@aliyun.com (G.L.); lilly_wang@126.com (B.W.); 171006165@qq.com (Y.Z.); cpulj@126.com (J.L.); abc_w@163.com (Q.W.); 2 Department of Chemical Defense Medicine, Faculty of Tropical Medicine and Public Health, Second Military Medical University, Shanghai 200433, China

**Keywords:** jellyfish, peroxiredoxin, antioxidant, cloning, ROS

## Abstract

We first identified and characterized a novel peroxiredoxin (Prx), designated as CcPrx4, from the cDNA library of the tentacle of the jellyfish *Cyanea capillata*. The full-length cDNA sequence of CcPrx4 consisted of 884 nucleotides with an open reading frame encoding a mature protein of 247 amino acids. It showed a significant homology to peroxiredoxin 4 (Prx4) with the highly conserved F-motif (^93^FTFVCPTEI^101^), hydrophobic region (^217^VCPAGW^222^), ^140^GGLG^143^ and ^239^YF^240^, indicating that it should be a new member of the Prx4 family. The deduced CcPrx4 protein had a calculated molecular mass of 27.2 kDa and an estimated isoelectric point of 6.3. Quantitative real-time PCR analysis showed that CcPrx4 mRNA could be detected in all the jellyfish tissues analyzed. CcPrx4 protein was cloned into the expression vector, pET-24a, and expressed in *Escherichia coli* Rosetta (DE3) pLysS. Recombinant CcPrx4 protein was purified by HisTrap High Performance chelating column chromatography and analyzed for its biological function. The results showed that the purified recombinant CcPrx4 protein manifested the ability to reduce hydrogen peroxide and protect supercoiled DNA from oxidative damage, suggesting that CcPrx4 protein may play an important role in protecting jellyfish from oxidative damage.

## 1. Introduction

Exposure to continuous environmental changes, such as solar radiation, pollution, microorganisms, pathogens, salinity and temperature, could lead to the activation of inner defense responses, including the production of reactive oxygen species (ROS) [1]. At low concentrations, ROS may facilitate processes, such as intracellular signaling and defense against microorganisms [2]. However, oxidative stress may occur in the case of excessive production and accumulation of ROS, which would result in a disturbance of metabolic balance, causing damage to cellular lipids, proteins and DNA [3,4]. It is already established that many organisms have both enzymatic and non-enzymatic antioxidant defense mechanisms to minimize such injuries. These antioxidants include various forms of peroxiredoxin, thioredoxin, catalase, glutathione peroxidase and superoxide dismutase [5].

Jellyfish (Scyphozoa) is a class of Cnidaria, which are abundant in pelagic oceanic waters. For the past few years, populations of jellyfish have been exploding in oceans around the world, which has led to many deleterious consequences, threatening human life, fisheries or even ecological balance. On the other hand, however, scientists have also discovered that there are many kinds of highly bioactive substances in the body of jellyfish, which may have good prospects for the development of new marine drugs [6,7,8]. In particular, as a representative of macroplankton, jellyfish are continuously exposed to harsh environmental factors, such as strong sunlight and ultraviolet (UV) radiation, which may lead to an increase in the production of ROS. Therefore, the exploration of the underlying molecular mechanisms that enable jellyfish to tolerate high levels of oxidative stress could help to better understand the impact of exposure to direct sunlight and UV radiation.

Scientists have isolated some proteins from the jellyfish, *Rhopilema esculentum* and *Stomolophus meleagris*, which have strong free radical scavenging abilities and can protect mouse skin lipid and collagen from UV radiation damage [9,10]. These results indicate that jellyfish have developed a wide range of powerful antioxidants for self-protection after long-term adaptive selection, suggesting that jellyfish are likely to be a natural resource of antioxidant and anti-UV radiation agents. However, the composition of the antioxidant system in jellyfish species and the sequences, expression levels and bioactivities of some important antioxidant enzymes have not yet been reported until now.

Peroxiredoxins (Prx proteins) are protective antioxidant enzymes, which are identified as a class of conserved proteins in many organisms, from yeast to mammals [11,12]. It is known that Prx proteins are classified into three types: typical 2-Cys Prx (Prx 1–4), atypical 2-Cys Prx (Prx 5) and 1-Cys Prx (Prx 6). The 2-Cys Prx proteins have two catalytically active Cys residues, termed the peroxidatic and the resolving Cys, whereas the 1-Cys Prx proteins have only the peroxidatic Cys. During the catalytic cycle, the peroxidatic Cys and the resolving Cys form a disulfide bond. In the typical 2-Cys Prx proteins, this bond is intermolecular, whereas in the atypical 2-Cys Prx proteins, it is intramolecular [13,14,15]. In recent years, the peroxiredoxin superfamily has become one of the hotspots in the research fields of antioxidants, and the most important functions of Prx is considered the scavenging of ROS. These proteins can also act as principal enzymes to regulate the intracellular H_2_O_2_ concentration [16]. Furthermore, Prx has been demonstrated to act as a signal peroxidase to receive, transduce and transmit peroxide signals in mammalian cells [17,18]. In spite of the isolation of Prx genes from numerous organisms, however, the expression and antioxidant function of Prx proteins still remains to be systematically investigated in the jellyfish species.

*Cyanea capillata* has a worldwide distribution and is one of the common kinds of jellyfish in the Southeast China Sea. In this study, we report a complete Prx cDNA, designated as CcPrx4, from *C. capillata.* We also characterize its tissue distribution, recombinant protein expression and antioxidant bioactivity *in vitro*. To our knowledge, this is the first report of a representative antioxidant enzyme from a jellyfish species.

## 2. Materials and Methods

2.1. cDNA Library Construction

Total RNA was extracted from the tentacle of *C. capillata* with TRIzol Reagent (Invitrogen, Carlsbad, CA, USA), and then, the mRNA was isolated using the Oligotex mRNA Spin-Column Kit (Qiagen, Valencia, CA, USA). The concentration of purified mRNA was determined at 260 nm using a BioPhotometer (Eppendorf, Hamburg, Germany). The cDNA library was constructed using the SMART cDNA Library Construction Kit (Clontech, Mountain View, CA, USA), according to the manufacturer’s instructions.

### 2.2. EST Analysis and Identification of CcPrx4

EST sequences obtained from the cDNA library of the *C. capillata* tentacle were compared with those in the GenBank database using the BLASTx program to identify genes encoding possible functional proteins. The BLASTx algorithm revealed that one of the EST sequences from the cDNA library showed a significant similarity to protein sequences of the Prx4 family. Thus, this EST sequence was chosen for further analysis and designated as CcPrx4. Subsequently, complete sequencing of both strands of CcPrx4 cDNA was carried out to confirm that it was a full-length cDNA.

### 2.3. Sequence Analysis of the Full-Length CcPrx4 cDNA

The similarity in nucleotide and protein sequences of CcPrx4 was analyzed using the BLAST algorithm [19,20]. The open reading frame (ORF) was determined using the ORF Finder program [21]. A multiple sequence alignment was conducted with the ClustalW2 program [22]. Conserved domains were analyzed using the InterProScan and CDD websites [23,24,25,26]. The signal peptide was predicted using the SignalP 4.1 Server [27,28]. The phylogenetic tree was constructed using the neighbor-joining (NJ) method with the MEGA 4 software package. Bootstrap trials were replicated 2000 times. The molecular mass and isoelectric point (pI) was determined using the ProtParam tool [29]. The secondary structure and three-dimensional modeling were predicted using the Phyre2 and SWISS-MODEL algorithms [30,31,32,33], respectively. PyMOL (version 0.99rc6 for Windows; Delano Scientific, San Carlos, CA, USA) was used to view and modify the image of the resulting three-dimensional model [34].

### 2.4. Quantification Analysis of CcPrx4 Expression by Quantitative Real-Time PCR

Total RNA was extracted using the UNIQ-10 Kit (Sangon Biotech, Shanghai, China) based on the manufacturer’s protocol. Then, single strand cDNA was synthesized according to the manufacturer’s instructions using the PrimeScript^®^ RT Reagent Kit (TaKaRa, Otsu, Shiga, Japan), with the total RNA as the template together with Random6 and Oligo (dT) primers. Two gene specific primers, CcPrx4-F (5ʹ-GCCAAGTTTATCCACAAGAGAC-3ʹ) and CcPrx4-R (5ʹ-ACTGCTTTTCCTTCCCAATGT-3ʹ), were designed to amplify a product of 103 bp. The *C. capillata* GAPDH gene (GenBank accession number KF595154), used as an internal control, was amplified using the gene specific primers, CcGAPDH-F (5ʹ-GGTGCCCATCAAAACATTATC-3ʹ) and CcGAPDH-R (5ʹ-GACACATCAGCAACTGGAACAC-3ʹ), that produced a fragment of 122 bp. Quantitative real-time PCR (qRT-PCR) was performed using an ABI PRISM 7300 Sequence Detection System (Applied Biosystems, Foster City, CA, USA). Each reaction (total volume: 25 μL) contained 0.2 μM each of the gene specific primers, 0.5 μL ROX Reference Dye, 12.5 μL SYBR^®^ Premix Ex Taq™ and 100 ng cDNA mix as the template, according to the manufacturer’s instruction for the SYBR^®^ Premix Ex Taq™ Kit (TaKaRa, Otsu, Shiga, Japan), and made up to a total reaction volume of 25 μL with diethyl pyrocarbonate (DEPC)-treated water. All treatments were performed in triplicate, and data were shown as the mean ± standard error (SE). The reaction used the thermal profile as follows: 95 °C for 30 s, followed by 40 cycles of amplification (95 °C for 15 s and 60 °C for 31 s). At the end of each PCR reaction, a dissociation curve was obtained by gradual heating of the PCR products from 60 to 95 °C to confirm that only one PCR product was amplified and detected. For both CcPrx4 and GAPDH internal control genes, there was only one peak present in the dissociation curves, indicating that the amplifications were specific. Relative gene expression was analyzed by the comparative Ct method (2^−ΔΔCt^ method) with GAPDH as the reference gene, and the results are presented as the relative quantity values [35]. Ct values for the CcPrx4 gene were standardized based on those for the GAPDH gene.

### 2.5. Construction of the Recombinant Plasmid CcPrx4/pET-24a

The coding region of CcPrx4 was amplified using standard PCR with the primers, 5ʹ-CTAGCTAGCATGAAAGATGACGAGTC-3ʹ and 5ʹ-CCGCTCGAGCATTTCTTCCTTC-3ʹ. The primers were designed with restriction enzyme sites for *Nhe* I and *Xho* I, respectively. The restriction sites are underlined in each primer. The PCR fragment and the pET-24a vector were separately digested with the selected restriction enzymes (NEB, Ipswich, MA, USA); then, the ligation was done at room temperature (25 °C) for 1 h using T4 DNA ligase (NEB, Ipswich, MA, USA). The ligated products were transformed into *Escherichia coli* TOP 10 competent cells (BioMed, Beijing, China). Positive recombinants were identified using agar plates containing 100 μg/mL kanamycin, followed by nucleotide sequencing of both strands to confirm in-frame insertion.

### 2.6. Expression and Purification of Recombinant CcPrx4 Protein in *E. coli*

The recombinant plasmid was transformed into the *E. coli* Rosetta (DE3) pLysS strain for protein expression. Transformed bacteria were propagated at 37 °C with shaking at 250 rpm in Luria-Bertani (LB) broth containing 100 μg/mL kanamycin and 34 μg/mL chloramphenicol. When the optical density (OD) of the bacteria at 600 nm reached 0.6, isopropyl-β-d-thiogalactoside (IPTG) was added at a final concentration of 0.5 mM. Then, the bacteria were shifted to the condition of 12 °C with shaking at 150 rpm to induce the production of the recombinant protein. After induction for 10 h, the bacteria were subjected to centrifugation at 12,000× *g* for 6 min, and the pellets were collected and resuspended in binding buffer (20 mM NaH_2_PO_4_, 500 mM NaCl, 30 mM imidazole, pH 7.4). Subsequently, the resuspended bacterial pellets were lysed by sonication in an ice bath, and the lysate was centrifuged at 12,000× *g* for 30 min at 4 °C. The supernatant was collected and applied to an ÄKTA protein purification system using a HisTrap High Performance (HP) chelating column (GE Healthcare, Milwaukee, WI, USA). The column was washed with binding buffer (20 mM NaH_2_PO_4_, 500 mM NaCl, 30 mM imidazole, pH 7.4), and then, the protein of interest was eluted from the column with elution buffer (20 mM NaH_2_PO_4_, 500 mM NaCl, 500 mM imidazole, pH 7.4). Samples collected from different steps were analyzed by 12% (*w/v*) sodium dodecyl sulfate-polyacrylamide gel electrophoresis (SDS-PAGE) based on the method of Laemmli, and the gel was stained with Coomassie blue R-250 [36]. Protein concentration was determined according to the method described by Bradford using a standard curve generated with bovine serum albumin (BSA) [37].

### 2.7. Western Blotting

After SDS-PAGE, the proteins in the gel were transferred to a polyvinylidene difluoride membrane (Millipore, Billerica, MA, USA). Subsequently, the membrane was incubated in blocking buffer 5% (w/v) fat-free milk powder in Tris-buffered saline and Tween 20 (TBST, containing 50 mM Tris, 150 mM NaCl, 0.05% (v/v) Tween 20, pH 7.6) with gentle shaking for 2 h at room temperature. It was then incubated with anti-His antibodies from mouse (1:2000 dilution, Tiangen, Beijing, China) at 4 °C overnight after rinsing the membrane with TBST three times. HRP-labeled goat anti-mouse IgG (Beyotime, Haimen, Jiangsu, China) diluted to 1:4000 was used as the secondary antibody. The G:BOX system (Syngene, Cambridge, UK) was used for chemiluminescent detection of cross-reacting proteins.

### 2.8. *In Vitro* Peroxidase Activity Assay

The reaction of Prx catalyzing the reduction of H_2_O_2_ with the presence of dithiothreitol (DTT) has been used to detect *in vitro* peroxidase activity [38]. The peroxidase activity of the purified CcPrx4 protein was evaluated as previously described, with little modification [39,40]. Briefly, 1-mL reaction mixtures, containing 50 mM 4-(2-hydroxyethyl)-1-piperazineethanesulphonic acid (HEPES) (pH 7.0), 5 mM DTT and the recombinant CcPrx4 protein or 100 μg/mL heat-inactivated recombinant CcPrx protein (control group), were incubated at room temperature for 10 min. H_2_O_2_ was added to a final concentration of 100 μM to initiate the reactions, and then, they were incubated for 0, 2.5, 5, 7.5 and 10 min at room temperature. Subsequently, 100 μL of 100% (w/v) trichloroacetic acid (TCA) was added to stop the reaction. The mixture was centrifuged to remove the precipitate, followed by the addition of 200 μL of 10 mM Fe(NH_4_)_2_(SO_4_)_2_ and 100 μL of 2.5 M potassium thiocyanate (KSCN), which could react with the remaining H_2_O_2_ and generated the red-colored ferrothiocyanate complex. The remaining amount of H_2_O_2_ in the mixture was estimated by measurement of the red ferrothiocyanate complex. The absorbance was measured at 475 nm. The clearance rate was calculated using the following formula: clearance rate = [(A_0_ − A*_x_*)/A_0_] × 100%, where A_0_ was the initial absorbance and A*_x_* was the absorbance after 2.5, 5, 7.5 and 10 min. The assay was performed in triplicate, and data were shown as the mean ± SE. Statistical analyses were carried out using IBM SPSS Statistics 19. The significance of the difference between each treatment group and the control was analyzed with one-way analysis of variance (ANOVA) and *p*-values lower than 0.05 were considered statistically significant.

### 2.9. Metal-Catalyzed Oxidation (MCO) Assay

The metal-catalyzed oxidation (MCO) assay was conducted to measure the potential of the purified CcPrx4 protein to protect supercoiled DNA against oxidative damage, according to the method described previously with slight modifications [41]. Fifty-microliter reaction mixtures containing 50 mM HEPES (pH 7.0), 35 μM FeCl_3_, 10 mM DTT, 1 μg supercoiled plasmid DNA of the pET-24a vector and CcPrx4 protein ranging from 25 to 200 μg/mL, were incubated at 37 °C for 2 h. At the end of the incubation, the reaction mixture was subjected to 1% (w/v) agarose gel electrophoresis containing Golden View™ (BioMed, Beijing, China) as the DNA stain to assess the DNA protection effect.

## 3. Results

### 3.1. Identification and Sequence Analysis of CcPrx4 cDNA

**Figure 1:**
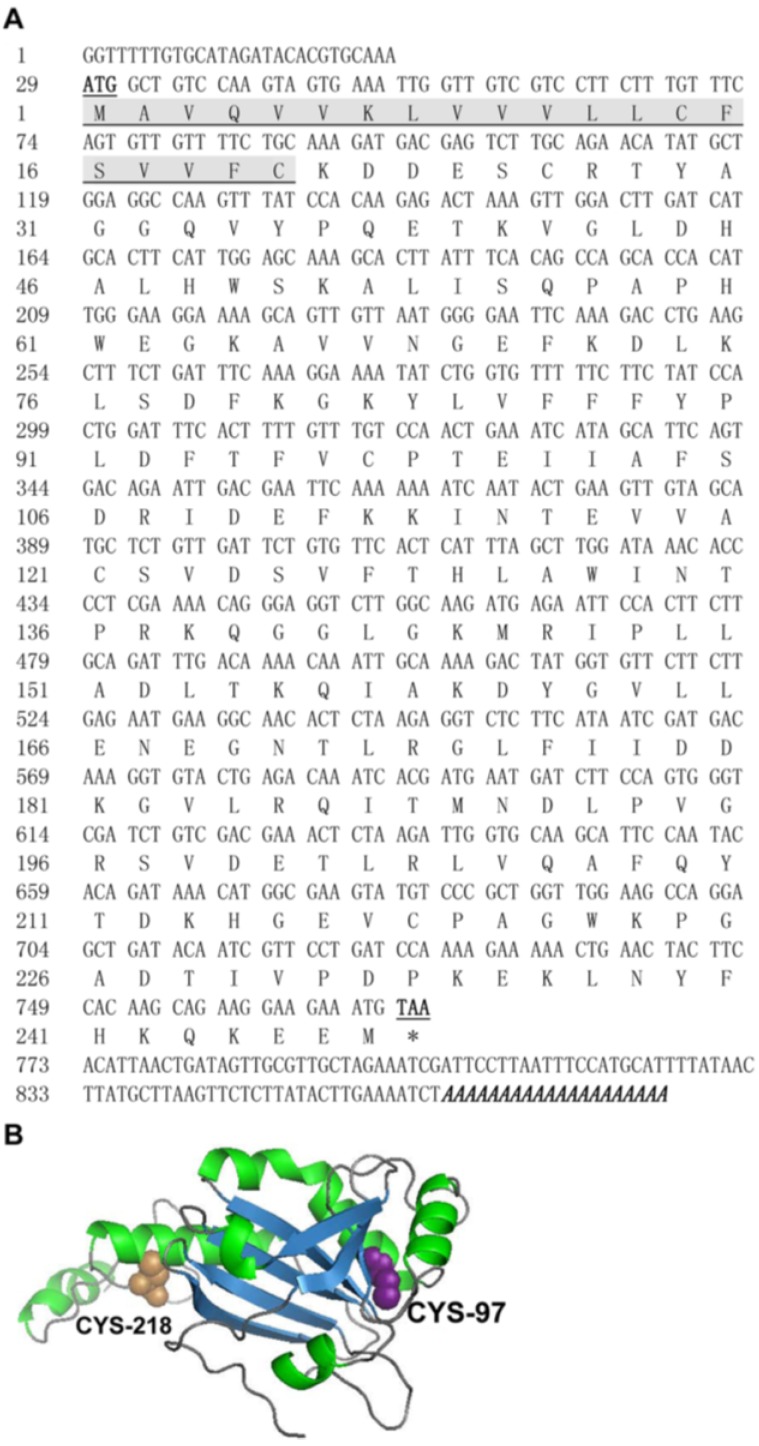
Sequence analysis of CcPrx4. (**A**) The full-length cDNA nucleotide and deduced amino acid sequences of CcPrx4. The signal peptide is underlined and shaded. The start (ATG) and stop (TAA) codons are bold and underlined. The poly (A) tail is shown bold and italicized at the end of the sequence. (**B**) The predicted three-dimensional structure of the CcPrx4 protein. Alpha-helices are shown in green, beta-strands in blue and beta-turns in grey. Balls in purple and copper represent CYS^97^ and CYS^218^, respectively.

An 884 bp full-length cDNA clone was directly isolated from a cDNA library of the *C. capillata* tentacle by large-scale random sequencing. As shown in Figure 1A, the cDNA contained a 28 bp 5ʹ-untranslated region (UTR), a single open reading frame (ORF) of 741 bp encoding a peptide of 247 amino acids and a 115 bp 3ʹ-UTR, including a stop codon (TAA) and a poly (A) tail. Homology analysis showed that this cDNA had high similarity with members of the Prx4 family. Therefore, the protein was provisionally identified as CcPrx4 (*C. capillata* peroxiredoxin4). The calculated molecular mass of the CcPrx4 protein was 27.2 kDa, with an estimated pI of 6.3. Further analysis revealed that the predicted CcPrx4 protein was a secreted protein, since a predicted *N*-terminal signal peptide with 20 amino acid residues was found in the deduced amino acid sequence. The tertiary structure of the CcPrx4 protein was also predicted using the Phyre2 program with the Prx4 protein from *Homo sapiens* (PDB ID: 3TKP, chain B) as the template. This human protein shared 65.0% identity with the CcPrx4 protein. The CcPrx4 protein consisted of eight alpha-helices and nine beta-strands, and the possible peroxidatic Cys^97^ was located at the front end of the fourth alpha-helix, while the possible resolving Cys^218^ was located in the middle of the eighth beta-strand (Figure 1B). The complete CcPrx4 cDNA sequence has been submitted to GenBank under the accession number KF201511.

### 3.2. Sequence Alignment and Phylogenetic Analysis of the CcPrx4 Protein

**Figure 2:**
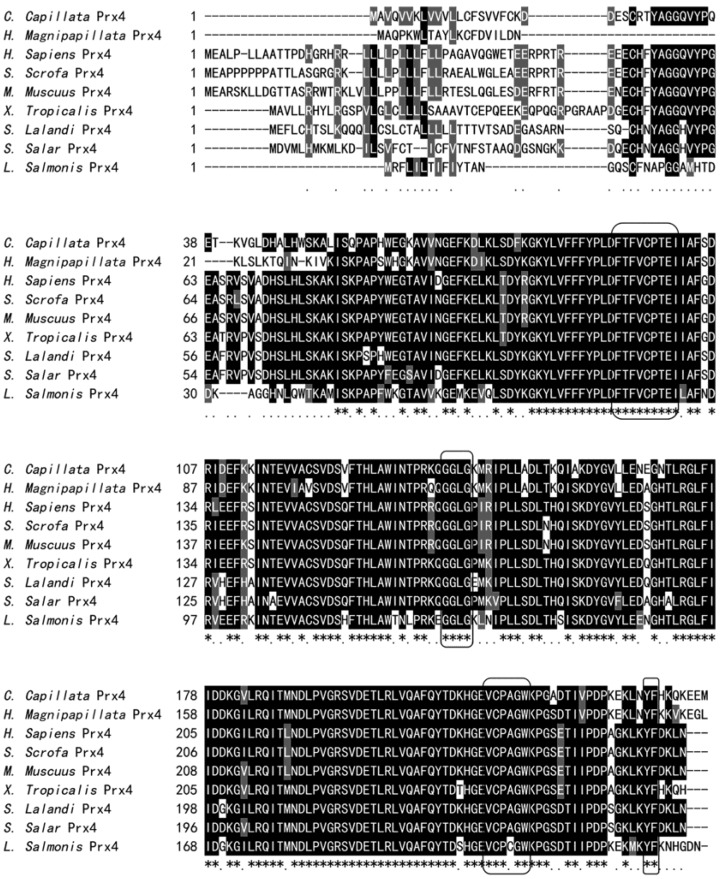
Multiple sequence alignment and phylogenetic analysis of the CcPrx4 protein. Multiple sequence alignment of the CcPrx4 amino acid sequence with other known Prx4 amino acid sequences from the GenBank database. Completely conserved residues across all the aligned sequences are shaded in black and marked with an asterisk (*) below. Highly conserved residues are indicated by dots (.) and shaded in gray. Absent amino acids are indicated by dashes (-). The conserved hydrophobic regions, F-motifs and GGLG and YF motifs are boxed. The common names of the organisms, the number of amino acids for each sequence and the GenBank accession numbers are indicated in Table 1.

As shown in Figure 2, the predicted amino acid sequence of the CcPrx4 protein displayed significant homology with other identified Prx4 proteins. The results demonstrated that the F-motif (^93^FTFVCPTEI^101^), hydrophobic region (^217^VCPAGW^222^) and ^140^GGLG^143^ and ^239^YF^240^ motifs were highly conserved among all the Prx4 proteins for the species analyzed. The two cysteine-containing motifs (^93^FTFVCPTEI^101^ and ^217^VCPAGW^222^) are believed to contain two highly conserved redox-active cysteines for the catalytic function of Prx. Therefore, Cys^97^ and Cys^218^ in the sequence of CcPrx4 protein might be the peroxidatic cysteine and the resolving cysteine, respectively [42,43]. The predicted protein also had the conserved ^140^GGLG^143^ and ^239^YF^240^ motifs, which are reported to serve as a keystone that strongly stabilizes the C-terminal structure [44]. Pairwise comparisons revealed that the Prx4 protein from *C. capillata* shared 64.4%–74.1% identity and 75.7%–81.0% similarity with the Prx4 proteins from other organisms, including vertebrates (human, *Homo sapiens*; mouse, *Mus mulatta*; pig, *Sus scrofa*; *etc.*) and invertebrates (sea louse, *Lepeophtheirus salmonis*; hydra, *Hydra magnipapillata*). Among these species, the CcPrx4 protein had the highest identity and similarity with the Prx4 protein from *H. magnipapillata*, which is also a marine invertebrate belonging to the phylum, Cnidaria (Table 1).

**Table 1:** The deduced amino acid sequence of the CcPrx4 protein compared with the Prx4 proteins from other species.

Species Name	Common Name	Accession Number	Sequence Size (aa)	Identity (%)	Similarity (%)
*C. capillata*	Jellyfish	KF201511	247	-	-
*H. magnipapillata*	Hydra	XP_004207404	247	74.1	81.0
*L. salmonis*	Sea louse	ACO12581	236	65.6	78.5
*M. musculus*	Mouse	NP_058044	274	66.4	76.2
*R. norvegicus*	Norway rat	NP_445964	273	65.2	75.7
*H. sapiens*	Human	NP_006397	271	65.0	77.0
*S. scrofa*	Pig	XP_001927404	272	64.4	76.7
*X. tropicalis*	Western clawed frog	NP_001006812	271	69.7	78.8
*S. lalandi*	Yellowtail kingfish	ACM47312	264	68.2	77.9
*S. salar*	Atlantic salmon	ACI69656	262	68.3	77.4

The accession numbers are from the GenBank database.

**Figure 3:**
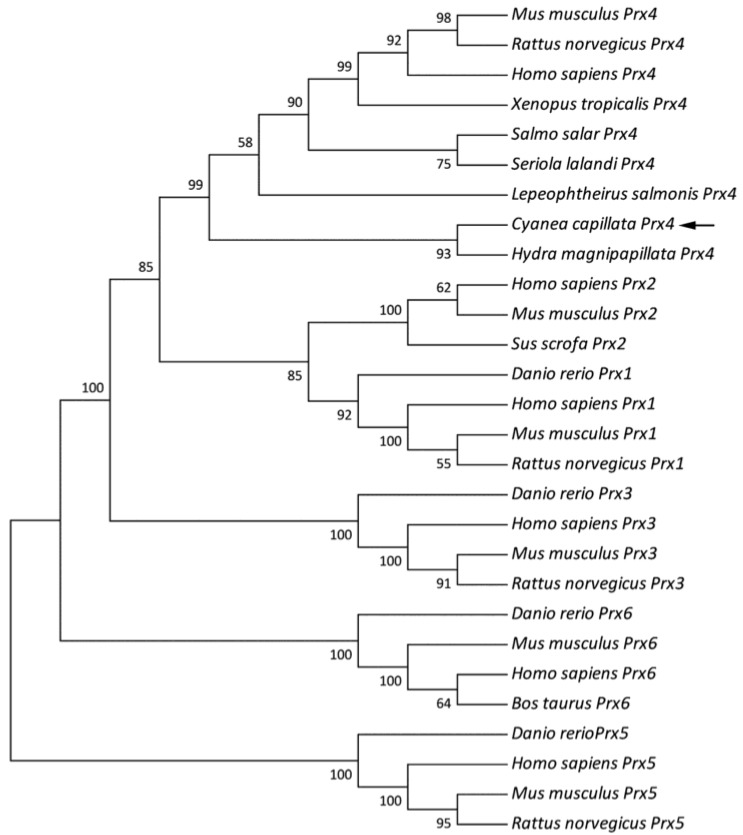
Phylogenetic analysis of the deduced amino acid sequence of the CcPrx4 protein compared with other known Prx proteins in the GenBank database. The number associated with each internal branch was the local bootstrap value, which was an indicator of bootstrap confidence.

In order to determine the evolutionary position of the CcPrx4 protein, a phylogenetic tree was constructed (Figure 3). In our phylogenetic tree, the 1-Cys Prx, typical 2-Cys Prx and atypical 2-Cys Prx subgroups were clustered distinctly. CcPrx4 was positioned in the Prx4 subgroup and most closely resembled the Prx4 from *H. magnipapillata*, which is another marine cnidarian (Class Hydrozoa). This grouping was well-supported by bootstrapping.

### 3.3. Tissue Distribution of CcPrx4

**Figure 4:**
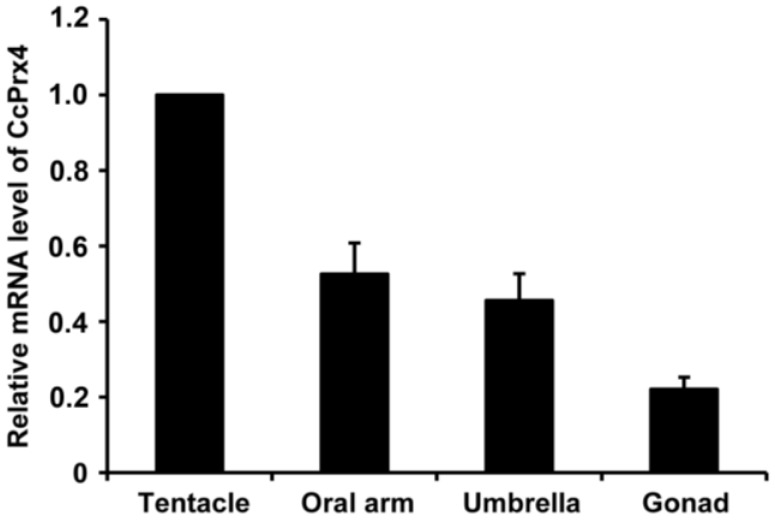
Tissue distribution of CcPrx4 mRNA. Relative expression was calculated using the 2^−ΔΔCT^ method with GAPDH as the reference gene, and the results are presented as the relative quantity values. All treatments were performed in triplicate, and data were presented as the mean ± SE (*n* = 3).

The CcPrx4 cDNA was originally cloned from a cDNA library made from *C. capillata* tentacle mRNA. Here, we have investigated the expression of CcPrx4 in other tissues of *C. capillata*. The relative tissue-specific expression was evaluated by comparing the transcript amount detected in each tissue with that in the tentacle. The results showed that CcPrx4 mRNA was detectable in all tissues analyzed. The tentacle tissue showed the highest level of expression, followed by the oral arm, the umbrella and, lastly, the gonad (Figure 4).

### 3.4. Recombinant Expression and Purification of the CcPrx4 Protein

**Figure 5:**
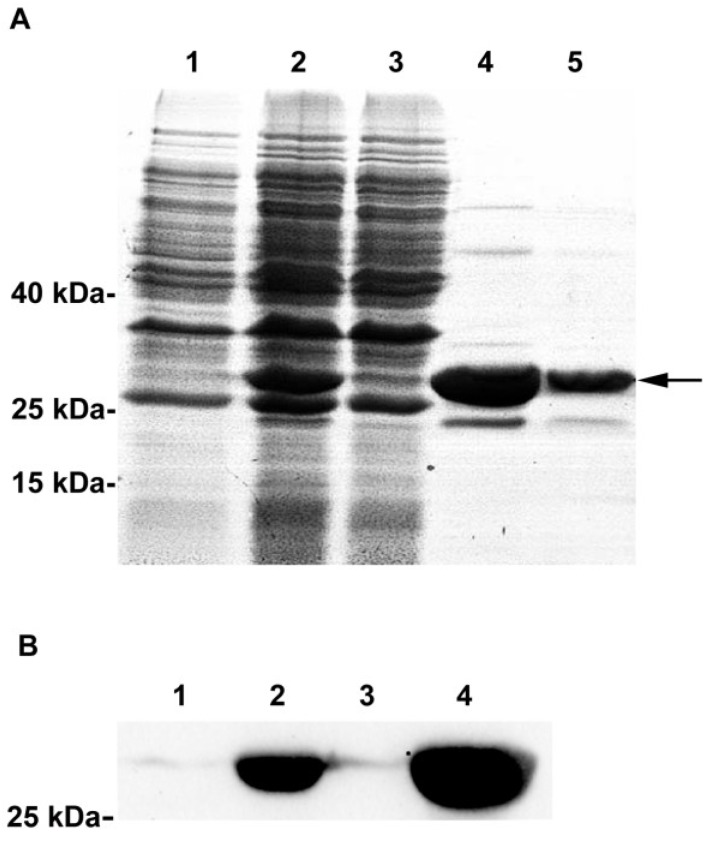
Expression and purification of the recombinant CcPrx4 protein. (**A**) 12% SDS-PAGE analysis of the samples collected from different steps of the expression and purification. Lane 1, whole cell lysates of recombinant *E. coli* Rosetta (DE3) pLysS before induction; lane 2, whole cell lysates of recombinant *E. coli* Rosetta (DE3) pLysS after induction with 0.5 mM isopropyl-β-d-thiogalactoside (IPTG) for 10 h at 12 °C; lane 3, fractions from the 30 mM imidazole wash of the HisTrap High Performance (HP) affinity column; lane 4, early fractions from the 500 mM imidazole elution of the HisTrap HP affinity column; lane 5, late fractions from the 500 mM imidazole elution of the HisTrap HP affinity column. The position corresponding to the recombinant CcPrx4 protein is indicated by an arrow. (**B**) Western blotting analysis of anti-His antibody cross-reactivity of the proteins separated by SDS-PAGE. The lanes are the same as described for SDS-PAGE in panel A.

A CcPrx4 cDNA encoding the mature protein (not including the signal peptide) was amplified from the *C. capillata* tentacle cDNA library and cloned into the pET-24a expression vector. The encoded protein was expressed in *E. coli* and then purified using column chromatography. As shown in Figure 5A, there was only one major protein and several very minor proteins that eluted from the column (lanes 4 and 5). The major protein was undetectable in the uninduced cells. The molecular weight (27.2 kDa) of the major protein corresponded well with the expected molecular weight of the recombinant CcPrx4 protein (26.1 kDa without the signal peptide, but with 1.1 kDa of His-Tag). Western blotting analysis using anti-His antibodies further confirmed that the major protein was the His-tagged CcPrx4 fusion protein (Figure 5B). Therefore, it is evident that the CcPrx4 protein was successfully expressed in *E. coli* and purified to a high level.

### 3.5. *In Vitro* Peroxidase Activity of the CcPrx4 Protein

**Figure 6:**
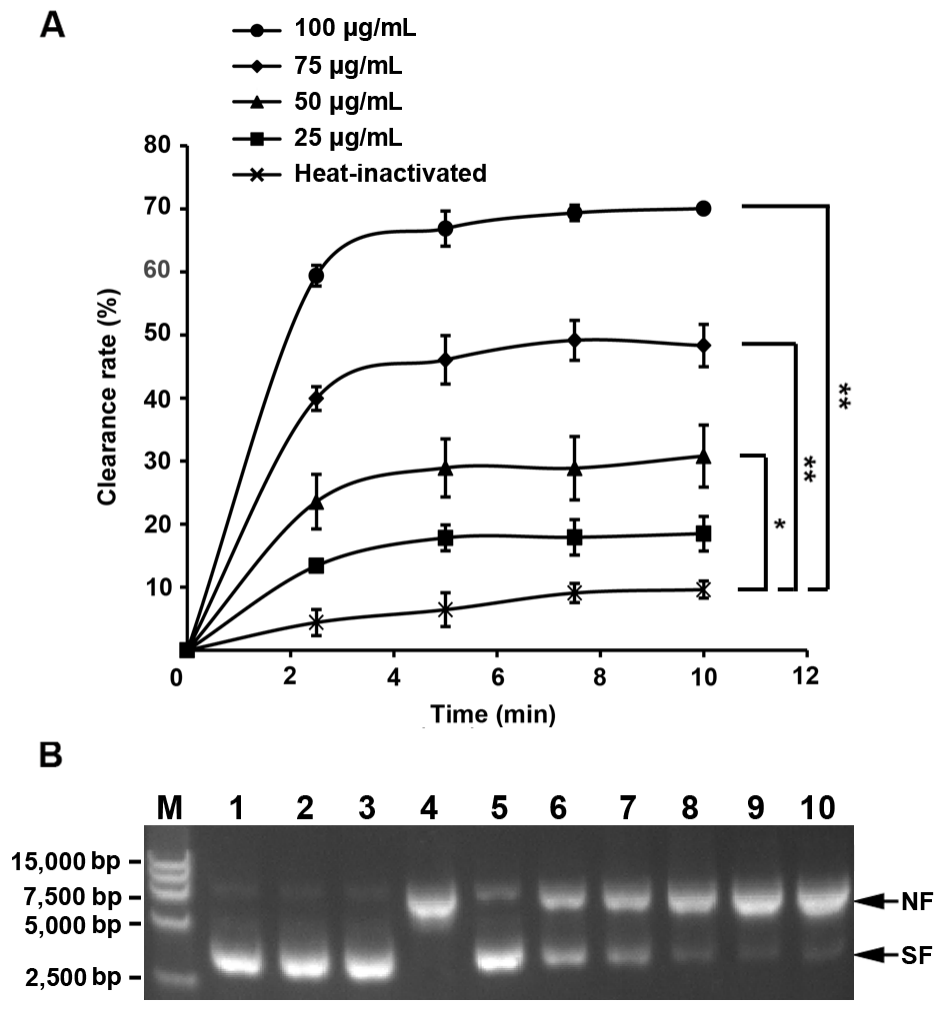
Biological function of the recombinant CcPrx4 protein. (**A**) *In vitro* peroxidase activity of the CcPrx4 protein in the presence of dithiothreitol (DTT). The clearance rate against reaction time (min) and protein concentration (μg/mL) was monitored to evaluate the H_2_O_2_ reduction activity of CcPrx4 protein. All treatments were performed in triplicate, and data were shown as the mean ± SE (*n* = 3, * *p* < 0.05 *vs.* the control (heat-inactivated), ** *p* < 0.01 *vs.* the control). (**B**) Effects of CcPrx4 protein in protecting the supercoiled structure of pET-24a plasmid DNA against oxidative damage in the metal-catalyzed oxidation (MCO) system. Lane 1, pET-24a plasmid DNA only; lane 2, pET-24a plasmid DNA and FeCl_3_; lane 3, pET-24a plasmid DNA and DTT; lane 4, pET-24a plasmid DNA, FeCl_3_ and DTT; lane 5–10, pET-24a plasmid DNA, FeCl_3_, DTT and different concentrations of the purified CcPrx4 protein (200, 150, 100, 75, 50 and 25 μg/mL, respectively). The bands corresponding to the nicked form (NF) and the supercoiled form (SF) of the plasmid DNA are indicated on the right-hand side.

As shown in Figure 6A, only very little degradation of H_2_O_2_ was observed with the heat-inactivated recombinant CcPrx4 protein (control group). However, the CcPrx4 protein distinctly displayed a time- and concentration-dependent activity to reduce H_2_O_2_. At 25 μg/mL of CcPrx4 protein, the clearance rate was slightly increased compared with the control group. When the protein concentration of CcPrx4 reached 75 and 100 μg/mL, the clearance rates were markedly increased to very high levels. It is known that the peroxidase activity of Prx is rapidly inactivated by hydrogen peroxide [45,46]. This is what could be occurring in the first 2.5 min of this assay. Before 2.5 min, the clearance rates were rapidly increased, suggesting that the reactions run very fast during the initial period. After 2.5 min, the reactions have entered a comparatively stationary process. These results suggested that CcPrx4 protein had a strong efficiency and speed to remove H_2_O_2_, thereby protecting jellyfish against oxidative stress.

### 3.6. Ability of the CcPrx4 Protein to Protect Supercoiled DNA

The metal-catalyzed oxidation (MCO) assay was performed in order to investigate the ability of CcPrx4 protein to protect the supercoiled structure of DNA against Fe^3+^ catalyzed oxidative damage [47]. The results showed that the pET-24a plasmid was not damaged by being incubated with FeCl_3_ or DTT separately at 37 °C for 2 h, while when incubated with FeCl_3_ and DTT simultaneously, the plasmid DNA was apparently converted from the supercoiled form to a nicked one. Furthermore, the amount of the nicked form of the plasmid decreased in a dose-dependent manner with increasing concentrations of the recombinant CcPrx4 protein (Figure 6B). Thus, this is evidence that the CcPrx4 protein plays a defensive role against DNA damage caused by metal-catalyzed oxidation.

## 4. Discussion

Jellyfish are members of the phylum, Cnidaria, and naturally distributed in temperate, subtropical and tropical waters. They are typified as free-swimming marine invertebrates consisting of a gelatinous umbrella-shaped bell and trailing tentacles. It has been demonstrated that high light and UV radiation may lead to an increase in the production of ROS, such as superoxide anion, hydrogen peroxide and hydroxyl radicals. The accumulation of these ROSs in cells can cause lipoperoxidation in membranes and DNA damage [48]. Therefore, the extreme conditions in which jellyfish live have attracted our attention, to try to discover the mechanisms that enable them to tolerate high levels of oxidative stress.

*C. capillata* is an off-shore jellyfish, which has a worldwide distribution and is very common in the coastal waters of southeast China. In the present study, an antioxidant enzyme gene, designated as CcPrx4, was isolated from the cDNA library made from the tentacle of *C. capillata*. Homology analysis showed that this novel molecule had a high similarity with the peroxiredoxin 4 family.

Peroxiredoxin, also called thioredoxin peroxidase (TPx), is a ubiquitous peroxide family, present both in prokaryotes and eukaryotes. It was first discovered as a new type of thiol-specific antioxidant protein from human red blood cells [40]. Many reports have indicated that peroxiredoxin can function to eliminate hydrogen peroxide and rapidly detoxify organic hydroperoxides (ROOH) and peroxynitrite (OONO^−^) [49,50]. Therefore, it is thought to be the principal enzyme for the removal of excessive H_2_O_2_ and to protect living organisms from such oxidative damages [51]. Moreover, peroxiredoxin also plays a key role in tumor suppression, immune response, signal transduction, as well as maintaining redox homeostasis [52,53,54,55,56,57,58]. Peroxiredoxins do not contain tightly bound metal ions, like other well-known antioxidant enzymes, but they contain highly conserved redox-active cysteine, which is involved in the catalytic mechanism [59].

So far, six kinds of Prx proteins (Prx1-6) have been reported that can be grouped into three categories: 1-Cys, typical 2-Cys and atypical 2-Cys, according to the number of the conserved cysteine residues and the formation of a disulfide bond between the two Cys during its catalytic cycle [13,14,15]. Multiple sequence alignment showed that the amino acid sequence of the CcPrx4 protein displayed significant homology with Prx4 proteins from other species, and that it exhibited structural features of the 2-Cys Prx family. The F-motif, including the peroxidatic cysteine, and the hydrophobic region, including the resolving cysteine, were both found in the CcPrx4 protein sequence, which might be required for its catalytic function, suggesting that this molecule cloned from jellyfish was capable of the same antioxidant attributes as other members of the 2-Cys Prx proteins. Meanwhile, the CcPrx4 protein contains an NH_2_-terminal hydrophobic region that is typical for the signal sequence of secreting proteins. Actually, Prx4 protein has been considered a secretory protein in mammals [60]. However, there is also a Prx4 protein from black tiger shrimp, *Penaeus monodon*, which does not possess an *N*-terminal signal peptide [61]. The phylogenetic tree revealed that CcPrx4 belonged to a Prx4 branch and most closely resembled the Prx4 from *H. magnipapillata,* another marine cnidarian. This result could be further supported by the pairwise alignment analysis, where CcPrx4 protein shared the highest identity and similarity with Prx4 protein from *H. magnipapillata*. Since *C. capillata* and *H. magnipapillata* are from the same phylum, they are relatively close to each other in evolutionary history and share similar structures.

Expression pattern analysis of CcPrx4 showed that it was found in all the tested jellyfish tissues, including tentacle, umbrella, oral arm and gonad. The ubiquitous expression of the CcPrx4 protein in jellyfish tissues indicates that it is a critical molecule that may potentially be involved in numerous physiological functions and can act as a very effective antioxidant to remove oxidative stress and protect jellyfish from damage by ROS.

We successfully constructed the recombinant plasmid, CcPrx4/pET-24a, and transformed it into the *E. coli* Rosetta (DE3) pLysS strain for protein expression and purification. SDS-PAGE and Western blotting results demonstrated that we had obtained the recombinant CcPrx4 protein with a very high purity. In the assay for *in vitro* peroxidase activity, the recombinant CcPrx4 protein could scavenge H_2_O_2_ and exhibited a concentration-dependent peroxidase activity, quickly scavenging H_2_O_2_ in about two minutes. The MCO system has been widely used to assess ROS damage to DNA and has been previously used to assay the antioxidant activity of Prx proteins from different subfamilies [62,63,64]. In this study, we found that the CcPrx4 protein could protect plasmid DNA from nicking. Moreover, this protective effect was apparently dose-dependent. When the concentration of the recombinant CcPrx4 protein reached 200 μg/mL, the formation of the nicked form was almost completely blocked. All these results indicate that CcPrx4 is a functional homologue of Prx4, which represents a potential protective barrier against oxidative damage in the body of jellyfish. As a result, we propose that the CcPrx4 protein might be a natural antioxidant and could be developed into the wide applications of food preservatives, sunscreens or drugs for the prevention and treatment of the diseases associated with oxidative stress.

## 5. Conclusions

In conclusion, here, we described the identification, cloning and strong antioxidant activities of a representative antioxidant enzyme from a jellyfish species. Our results strongly support that the CcPrx4 protein is a key component of the antioxidant system of the jellyfish, *C. capillata*, which protects the jellyfish body against oxidative exposure. As far as we know, it is the first full-length antioxidant enzyme gene isolated and characterized from jellyfish, which provides a scientific foundation for understanding the mechanism that enables jellyfish to tolerate high levels of oxidative stress and for developing new types of marine drugs.

## Abbreviations

BLAST: Basic Local Alignment Search Tool
CDD: Conserved Domain Database
MEGA: Molecular Evolutionary Genetics Analysis
ROX: Carboxy-X-rhodamine
SYBR: Synergy Brands
Ct: cycle threshold
TOP 10: dh10 β *Escherichia coli* strain
HRP: horse radish peroxidase
G:BOX: Syngene system for fluorescence and visible applications
PDB: Protein Data Bank
YF motif: Tyr-Phe motif
